# Evaluation of median urinary iodine concentration cut-off for defining iodine deficiency in pregnant women after a long term USI in China

**DOI:** 10.1186/s12986-019-0381-4

**Published:** 2019-09-09

**Authors:** Huidi Zhang, Meng Wu, Lichen Yang, Jinghuan Wu, Yichun Hu, Jianhua Han, Yunyou Gu, Xiuwei Li, Haiyan Wang, Liangkun Ma, Xiaoguang Yang

**Affiliations:** 1 The Key Laboratory of Trace Element Nutrition, National Institute for Nutrition and Health, Chinese Center for Disease Control and Prevention, 29 Nan Wei Road, Xicheng District Beijing, China; 2 Shaanxi Provincial Centre for Disease Control and Prevention, No.3, jiandong street, Xi’an, Shaanxi China; 3 The Department of Laboratory Medicine, Peking Union Medical College Hospital, Chinese Academic Medical Science and Peking Union College, No.1 Shuaifuyuan Wangfujing, Dongcheng District Beijing, China; 4 The National IDD Reference Laboratory, National Institute for Nutrition and Health, Chinese Center for Disease Control and Prevention, 29 Nan Wei Road, Xicheng District Beijing, China; 5 Department of Obstetrics-gynecology, Peking Union Medical College Hospital, No.1 Shuaifuyuan Wangfujing, Dongcheng District Beijing, China

**Keywords:** Urinary iodine, Chinese pregnant woman, Thyroid function

## Abstract

**Background:**

The WHO/UNICEF/ICCIDD define iodine deficiency during pregnancy as median urinary iodine concentration (MUIC) ≤ 150 μg/L. China implemented universal salt iodization (USI) in 1995, and recent surveillance showed nationwide elimination of iodine deficiency disorders (IDD). Data from 2014 showed that the MUIC in 19,500 pregnant women was 154.6 μg/L and 145 μg/L in 9000 pregnant women in 2015. However, symptoms of iodine deficiency were absent. Our study sought to evaluate whether MUIC below 150 μg/L affects thyroid function of Chinese pregnant women and their newborns in Chinese context.

**Methods:**

We screened 103 women with normal thyroid function and MUIC lower than 150 μg/L during week 6 of pregnancy at Peking Union Medical College Hospital. Patient demographics and dietary salt intake were recorded. Subjects were followed at 12, 24, and 32 gestational weeks. At each visit, a 3-day dietary record, drinking water samples, and edible salt samples were collected and analyzed for total dietary iodine intake. Additionally, 24-h urine iodine and creatinine were measured. Blood tests assessed thyroid function in both mothers and newborns.

**Results:**

Of 103 pregnant women enrolled, 79 completed all follow-up visits. Most subjects maintained normal thyroid function throughout pregnancy. However, 19 had thyroid dysfunction based on thyroid stimulating hormone and free thyroxine levels. The median serum iodine was 71 μg/L (95% CI: 44, 109). The median thyroglobulin was < 13 μg/L. values above this level indicate iodine deficiency in pregnant women. The median dietary iodine intake during pregnancy, derived from the 3-day record and measures of water and salt, was 231.17 μg/d. Assuming 90% urinary iodine excretion (UIE), 200.11 μg/d UIE means the 222.34 μg iodine loss per day, suggesting that subjects had a positive iodine balance throughout pregnancy. All neonatal blood samples showed TSH levels lower than 10 mIU/L, indicating normal thyroid function. No significant difference was found among gestational weeks for urinary iodine, and the MUIC in subjects who completed 3 follow-up visits was 107.41 μg/L.

**Conclusion:**

Twenty years after implementing USI, expectant Chinese mothers with MUIC of 107.4 μg/L, less than the WHO’s 150 μg/L benchmark, maintained thyroid function in both themselves and their newborn babies.

## Background

Iodine, one of the essential trace elements in the body, is required for the production of thyroid hormones, which are necessary for fetal cognitive development. Severe iodine deficiency during pregnancy can cause irreversible damage to the nervous system and intelligence of the fetus [1]. Iodine status in pregnancy is a focus of several current studies. The median urinary iodine concentration (MUIC) is a commonly used indicator to assess iodine status because the majority of iodine is excreted in urine [2, 3]. A joint task force of the World Health Organization (WHO), the United Nations Children’s Fund (UNICEF), and the International Council for the Control of Iodine Deficiency Disorders (ICCIDD) [4] recommends that the MUIC of pregnant women be between 150 and 249 μg/L. MUIC less than 150 μg/L has been defined as iodine deficiency.

China once had one of the world’s greatest rates of iodine deficiency diseases (IDD). In 1995, a program of universal salt iodization (USI) was introduced to eradicate the deficiency [5]. Since then, the iodine nutrition level of Chinese residents has improved. IDD reached the level of sustained elimination in 2000 [6] and was eliminated on the national level as of the 2014 surveillance. The latest Chinese monitoring data showed that the iodine nutritional status of the general population, especially in children ages 8–10 years old, has already reached the appropriate level [7]. But according to the 2015 Chinese Adult Chronic Disease and Nutritional Surveillance (CACDNS), the MUIC of pregnant women in more than 50% of the provinces was less than 150 μg/L (unpublished). This study involved 9000 pregnant women with a MUIC of 145 μg/L, yet mothers in this study did not exhibit symptoms of iodine deficiency or thyroid dysfunction. Therefore, determining whether MUIC < 150 μg/L affects thyroid function in pregnant women and their newborns is of interest.

The aim of this study was to evaluate the thyroid function of Chinese pregnant women with MUIC below 150 μg/L throughout gestation. Additionally, our results may potentially provide data to support the salt iodization policy in China.

### Hypothesis

In this study, we postulated two things: 1) if thyroid function during each trimester of pregnancy is normal, the total iodine intake is positively balanced compared with the excretion of urine iodine, and 2) if the thyroid stimulating hormone (TSH) of the newborn is normal, the mothers’ iodine nutritional status during pregnancy is at a satisfactory level. MUIC of less than 150 μg/L in pregnant women may not cause compromised thyroid function in either women or newborns in this China population.

## Methods

### Procedures

**Fig. 1:**
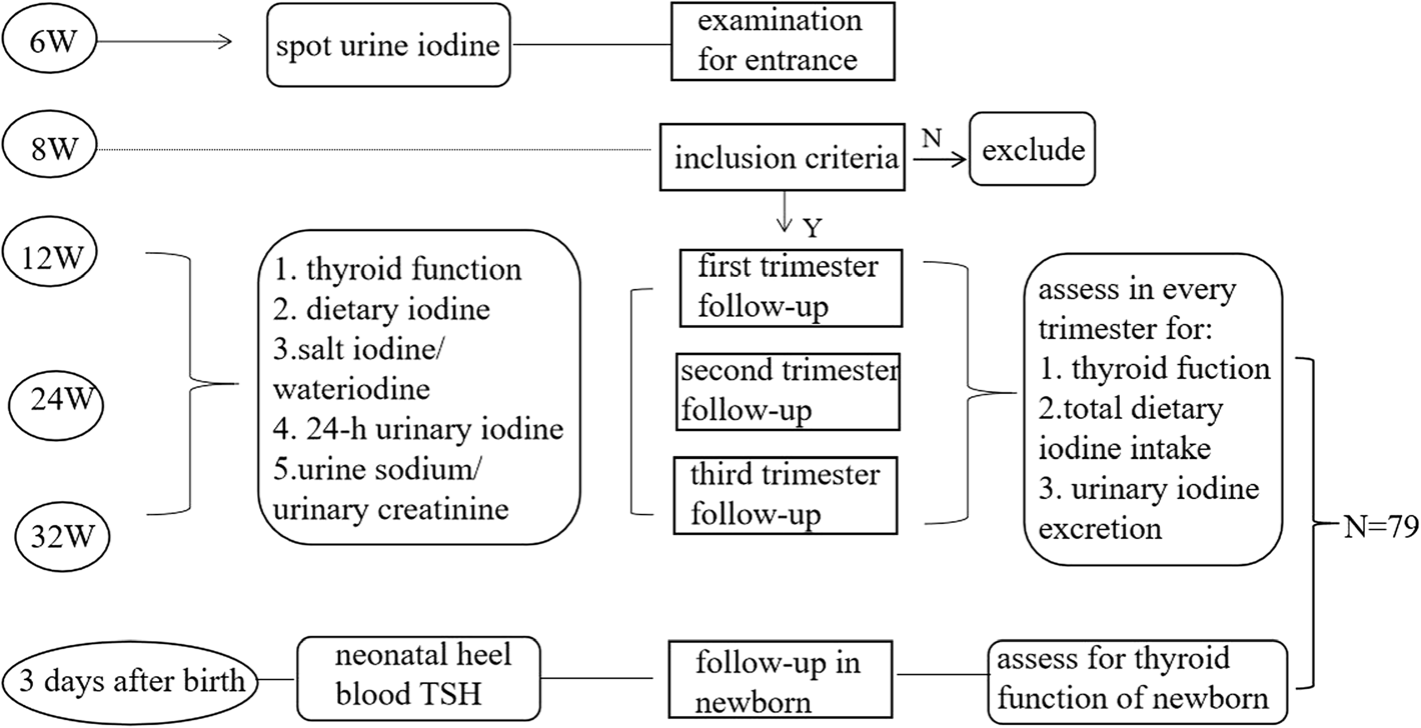
The technology roadmap of the study

To verify our hypothesis, we carried out a study in Beijing Peking Union Medical College Hospital. Subjects were enrolled at the first antenatal visit at the 6th week of pregnancy. To verify the current threshold of 150 μg/L MUIC recommended by WHO/UNICEF/ICCIDD [4] and to test our hypothesis, volunteers were enrolled if they had normal thyroid function. This included negative thyroglobulin antibody (TgAb) and thyroid peroxidase antibody (TPOAb) but apparent iodine deficiency, based on morning spot urine iodine concentration lower than 150 μg/L. During routine examinations in the 12th, 24th, and 32nd gestational weeks, thyroid function (including free triiodothyronine (FT3), free thyroxine (FT4), TSH, thyroid globulin (Tg) and serum iodine (SI)), dietary iodine intake, and 24-h urinary iodine and creatinine excretion were assessed. Following the birth of the child, neonatal heel blood was also tested for TSH to evaluate the thyroid function of the newborn. The schedule of visits is detailed in Fig. 1.

Because of large physiological fluctuations in thyroid biomarkers and lack of clear definition of “normal” thyroid function during pregnancy, we consulted an endocrinologist for subjects having a one-time abnormal thyroid function test. If the doctor determined that medical intervention for thyroid dysfunction was not indicated and thyroid testing after the next visit was normal, the subject could still be included in our analysis.

### Participants

Subjects were recruited from the Obstetric Clinic of Peking Union Medical College Hospital between June 2016 and May 2017. Basic demographic information such as name, age, ID number, and contact method, was collected. Inclusion criteria included a spot urinary iodine concentration less than 150 μg/L, TSH lower than 2.5 mIU/L, and negative TPOAb and TgAb. Additionally, the women had to be pregnant with a single fetus in good physical condition. These women were enrolled following the informed consent and ethical review. The exclusion criteria were as follows: (1) subjects who had personal history and family history of thyroid disease; (2) subjects with a visible or palpable goiter; (3) subjects who took thyroid-related medicine; and (4) pregnant women who had some other gynecologic condition, such as uterine fibroids. In order to improve the compliance of our subjects, we set up free lectures to explain the related health care knowledge of pregnancy while also emphasizing the importance of dietary records and drinking water/edible salt sample collection in each trimester.

### Blood sample collection and analysis

Venous blood samples to assess thyroid function and iodine status were collected during routine antenatal care and included FT3, FT4, TSH, Tg, SI, TgAb, and TPOAb. Serum FT3, FT4, and TSH were measured by an automatic chemiluminescence immunoanalyzer (Centaur XP, Siemens, Germany). Tg, TgAb, and TPOAb were tested using an automatic immune analyzer (Cobas, Roche Diagnostics, Tokyo, Japan), and SI was detected by inductively coupled plasma mass spectrometry (ICP-MS; Thermo Fisher Scientific, Waltham, MA). These analyses were performed at the laboratory department of Beijing Peking Union Medical College Hospital. Diagnostic criteria for abnormal thyroid function are shown in Table 1.

**Table 1:** Thyroid function in relation to plasma levels of TSH and FT4

Thyroid function	TSH (mIU/L)^a^	FT4 (pmol/L)^b^
Normal thyroid function	0.1–3	10.42–24.32
Isolated hypothyroxinemia	0.1–3	< 10.42
Subclinical hypothyroidism	> 3	10.42–24.32
Clinical hypothyroidism	> 3	< 10.42
Subclinical hyperthyroidism	< 0.1	10.42–24.32
Clinical hyperthyroidism	< 0.1	> 24.32

^a^Normal range for TSH (mIU/L) [8]: 0.13–3.93 (T1), 0.26–3.50 (T2), and 0.42–3.85 (T3)

^b^Normal range for FT4 (pmol/L) 10.42–24.32 pmol/L referred the value of clinical practice in Peking Union Medical College Hospital

Newborn TSH, obtained from heel blood samples collected 3 to 4 days after birth, is also a sensitive indicator of iodine deficiency in pregnancy [9, 10]. This work is part of the Beijing Neonatal Disease Screening Center.

### 24-h urine collection and analysis

Following enrollment, all participants received detailed instruction for the 24-h urine collection. From the 12th gestational week, they were asked to collect the 24-h urine the day before their routine prenatal examination. After complete remixing in the urine bag, participants accurately measured and recorded the total volume of urine collected using the measuring cups provided. Subjects then aliquoted the remixed urine into two 50 mL tubes which were kept and sent to the hospital for further assessment. If the urine samples could not be sent for analysis in a timely manner, they were stored at 4 °C. One tube was tested by the Laboratory of Beijing Peking Union Medical College Hospital for the detection of sodium content and creatinine (AU7200, Beckman Coulter, Brea, CA). The other tube of urine was transported to the National Iodine Deficiency Disease Reference Laboratory for measurement of urinary iodine concentration by As-Ce catalysis spectrophotometry, the gold standard method for estimating iodine content in salt [11]. Urinary iodine level was tested as described, and the 24-h urinary iodine excretion was calculated by urinary iodine concentration × urinary volume.

### Dietary total iodine analysis and sample collection

Total dietary iodine was measured from food, iodized salt, and water. Three days before each routine examination, participants were asked to record all foods, soups, and drinking water in a 3-day dietary record. An electronic dietary scale was provided to each subject to weigh all foods and water. Trained health workers answered any questions and checked the dietary records on the day of clinical follow-up. The iodine content in food was calculated using reference values from the China Food Composition database (2009) and our laboratory database. In addition, participants collected 20 g of edible salt and 50 mL of drinking water for analysis of salt and water iodine content. This testing was carried out by the National IDD Reference Laboratory.

The calculation for edible salt iodine intake was salt intake × salt iodine content × (1 – the loss rate of cooking). We adopted a cooking loss rate of 20%, which is recommended by the WHO/UNICEF/ICCIDD [4]. The salt intake was calculated by the standard method of 24-h urine sodium content (mmol) × molar mass of sodium chloride (g/mol) / 1000. Twenty-four-hour urine sodium excretion has become the preferred method of obtaining data on salt intake in population surveys [11].

The actual amount of drinking water was recorded, and the amount of soup was calculated from the 3-day dietary record. Water iodine intake of each pregnant woman was calculated via this formula: the sum of drinking water and soups (mL) × the iodine content of water / mL.

### Statistical analysis

General statistical analysis was performed using SAS 9.4 (SAS Institute, Cary, NC). A *P*-value less than 0.05 was considered statistically significant. The normality distribution tests were performed on all the data. If the indices were not normally distributed, they were presented as a median (P2.5, P97.5). The others, which did follow normal distributions, were expressed as mean ± standard deviations ($\overline{X}$ ± SD). This study adopted the normal percentile method to formulate the reference range for some indices, and it could be represented by P2.5–P97.5. Because urinary iodine concentration (UIC) and thyroid function may be affected by some factors more than others, we conducted a one-way analysis of variance (ANOVA) to analyze the influence of other factors. The comparison of constituent ratio and rate was carried out using Chi-squared test. In order to explore changes in thyroid function at several gestations, the indices were analyzed by variance analysis of repeated measurement starting from 6 weeks’ gestation. Pearson’s Product-Moment Coefficient was employed to examine the correlation of indices that followed a normal distribution. Spearman’s rank correlation coefficient was used to analyze the association between other indicators which did not obey a normal distribution.

## Results

### Baseline characteristics of subjects

A total of 103 pregnant women were enrolled in this study, and 79 finished all 3 follow-up visits. Twenty-four participants were lost to follow-up for a variety of reasons, such as inconvenience of the 24-h urine collection and 3-day dietary record or after refusing the blood draw for thyroid function monitoring. There was no significant difference between those who completed all visits and those lost to follow-up, as analyzed through baseline characteristics. Having subjects lost to follow-up did not impact the analysis in this study.

After screening for inclusion criteria, we obtained the urinary iodine distribution of the satisfactory samples. No significant difference was found between UIC and thyroid function according to different demographic factors, including age, nationality, education, occupation, and income. The UIC at 6 weeks’ gestation had a normal distribution with a mean of 85 μg/L. The TSH and FT4 were in the normal range with the average level of these two indices being 1.45 mIU/L and 15.76 pmol/L, respectively.

### Distribution and assessment of thyroid function in different trimesters

Our cohort of 79 pregnant women each underwent thyroid function tests 4 times during the study. There were significant differences among serum FT3, FT4, TSH, SI, and Tg across trimesters (Table 2). The FT3 and FT4 of pregnant women at 3 follow-up visits were significantly different compared with the values from the 6th gestational week (*P* < 0.05). Starting from 6 weeks’ gestation, the level of FT3 and FT4 decreased gradually, and turned to be smooth at 24th weeks. The levels of TSH first decreased and then rose to a “U” shape. Comparison of TSH in any trimester with the former period, found that it was significantly different. SI also decreased with time; when compared with the first trimester, the 24th and 32nd weeks were significantly different. With respect to FT3 and FT4, although there was a significant difference between 12th and the 24th and 32nd weeks, there was no significant difference between the 24th and 32nd weeks. Similar to TSH, Tg also had a U-shaped curve. The value of Tg in the 12th, 24th, 32nd gestational weeks was 8.91 μg/L, 7.74 μg/L, and 9.92 μg/L, respectively.

**Table 2:** Comparison of Thyroid function parameters in different gestation

Gestation	N	FT3 (pmol/L)	FT4 (pmol/L)	TSH (mIU/L)	SI (μg/L)	TG (μg/L)
6th	79	4.74 ± 0.52	16.01 ± 2.00	1.48 ± 0.65	–	–
12th	79	4.44 ± 0.56^a^	15.38 ± 1.97^a^	1.08 ± 0.70^a^	80.11 ± 15.72	8.91(6.83~15.22)
24th	78	3.90 ± 0.36^ab^	12.59 ± 1.51^ab^	1.58 ± 0.63^b^	68.99 ± 13.39^b^	7.74(5.03~12.98)^b^
32th	78	3.94 ± 0.78^ab^	12.62 ± 1.84^ab^	1.74 ± 0.73^abc^	69.14 ± 15.75^b^	9.92(6.19~16.33)^c^
F		50.36	132.61	32.61	38.39	14.24
*P* value		< 0.0001	< 0.0001	< 0.0001	< 0.0001	< 0.0001

*FT3* Free Triiodothyronine, *FT4* Free Thyroxine, *TSH* Thyroid Stimulating Hormone, *SI* serum iodine, *TG* thyroid globulin. ^a^*p* < 0.05 between each trimesters with the 6th weeks; ^b^*p* < 0.05 between each trimesters with the 12th weeks; ^c^*p* < 0.05 between each trimesters with the 24th weeks

At 6 weeks’ gestation, all 79 pregnant women had normal thyroid function (Table 3). During pregnancy, 19 subjects experienced thyroid dysfunction once, and 1 subject had it twice. The rate of thyroid dysfunction in 12th, 24th, and 32nd gestational weeks was 8.9, 6.4, and 11.5%, respectively. There was no significant difference in thyroid function by trimester (*P* > 0.05).

**Table 3:** Prevalence of thyroid functions according to iodine status

Gestation	N	Normal thyroid function	Clinical hypothyroidism	Subclinical hypothyroidism	Clinical hyperthyroidism	Subclinical hyperthyroidism	Isolated hypothyroxinemia
6th	79	79 (100%)	0	0	0	0	0
12th	79	72 (91.14%)	0	2 (2.53%)	1 (1.27%)	4 (5.06%)	0
24th	78	73 (93.59%)	1 (1.28%)	0	0	0	4 (5.13%)
32th	78	69 (88.46%)	1 (1.28%)	4 (5.13%)		1 (1.28%)	3 (3.85%)
Total	314	293 (93.31%)	2 (0.64%)	6 (1.91%)	1 (0.032%)	5 (1.59%)	7 (2.23%)

### Total dietary iodine intake during the entire pregnancy

A total of 237 samples of drinking water and edible salt were collected during the 3 follow-up visits (Table 4). The median (inter-quartile range (IQR) water iodine content was 2.0 μg/L (0.4–2.9 μg/L). There was no difference in water iodine content from different sources such as tap water, bottled water, or filtered water. The amount of water intake included drinking water and soup. There was no significant difference in water iodine intake throughout the pregnancy: 3.12 μg/d, 3.58 μg/d and 3.23 μg/d, in the 1st, 2nd, and 3rd trimesters, respectively.

**Table 4:** Comparison of total dietary iodine intake in different gestation (μg/d)

Gestation	N	Water iodine intake	Food iodine intake	Edible salt intake	Total iodine intake
12th	79	3.12^a^ (0.63~5.23)	59.62 (41.91~116.71)	140.04 (111.52~173.58)	218.14 (177.91~306.58)
24th	79	3.58 (1.05~6.12)	63.22 (42.52~104.35)	153.25 (126.96~216.63)	233.51 (184.04~345.74)
32th	79	3.23 (0.42~5.79)	62.51 (44.52~114.28)	163.09 (116.40~206.30)	243.66 (194.11~328.62)
F		1.12	0.33	2.75	0.22
P value		0.3092	0.6954	0.0674	0.7996
Total		3.24	62.22	152.03	231.17

^a^Median (inter-quartile range)

The median (IQR) iodine content of edible salt was measured as 22.84 mg/kg (21.15–25.49 mg/kg). According to the results, the coverage rate of iodized salt was 100% and edible rate of adequately iodized salt was 94% (In this study, 25 mg/kg iodine in edible salt in Beijing was adopted as the standard from China’s standard [GB 26878–2011], the allowance range was 18–33 mg/kg). The mean value of 24-h urine sodium content was 144.96 mmol, and the salt intake in this study was calculated as 8.48 g/d. Therefore, the average total salt iodine intake during the 3 trimesters was as follows: 140.04 μg/d, 153.25 μg/d, and 163.09 μg/d.

The food iodine intake in each trimester was 59.62 μg/d, 63.22 μg/d, and 62.51 μg/d, respectively. The total dietary iodine intake was 218.14 μg/d in the 1st trimester, 233.51 μg/d in the 2nd, and 243.66 μg/d in the 3rd. There was no significant difference in the intake of food iodine, edible salt iodine, and total dietary iodine at different stages of pregnancy.

### Distribution and comparison of urine indices

The median volume of 24-h urine was 1863 mL (Table 5). The MUIC and urinary iodine excretion (UIE) of all participants were 107.41 μg/L (IQR 83.84–150.13 μg/L) and 200.11 μg/d (IQR 152.75–265.50 μg/d), respectively. There were no significant differences in these three indicators between the trimesters.

**Table 5:** Urinary iodine and urinary creatinine during different pregnancy

Gestation	N	V24 h (ml)	MUIC (μg/L)	UIE (μg/d)	MUC (g/L)	UCE (g/d)	UICr (μg/g)
Total	237	1863 (1450~2395)	107.41 (83.84~150.13)	200.11 (152.75~265.50)	0.54 (0.43~0.72)	1.06 (0.90~1.24)	195.70 (102.81~146.65)
12th	79	1850 (1425~2215)	101.56 (83.18~140.32)	185.00 (142.75~254.76)	0.55 (0.44~0.69)	1.02 (0.94~1.18)	188.26 (144.81~256.74)
24th	79	1900 (1450~2500)	108.00 (85.14~137.00)	209.10 (153.88~270.00)	0.53 (0.40~0.69)	1.05 (0.85~1.24)	204.88 (160.39~272.21)
32th	79	1900 (1400~2300)	113.00 (80.94~157.00)	198.5 (161.28~274.00)	0.58^b^ (0.45~0.88)	1.08^ab^ (0.92~1.32)	195.70 (142.80~233.94)
F		1.69	1.16	1.82	3.62	5.39	1.82
P value		0.1878	0.3149	0.1652	0.03	0.01	0.17

^a^*p* < 0.05 between each trimesters with the 12th weeks; ^b^*p* < 0.05 between each trimesters with the 24th weeks;V24 h, 24-h urine volume; MUIC, median urinary iodine concentration; UIE, urinary iodine excretion; MUC, median urine creatinine; UCE, median urine creatinine excretion; UICr, creatinine-adjusted urine iodine

Because 24-h urine is not easy to collect, we usually measure the content of creatinine to correct for its influence on the UIE. The median urine creatinine excretion (UCE) was nearly 1 g/d, and the amount of UCE increased throughout gestation (*P* < 0.01), which was consistent with other reports [11]. Creatinine was used as a correction factor to minimize the variation in urine volume for its relatively constant excretion rate [12]. These data also demonstrated the good quantity of the 24-h urine collection in our study. On the other hand, the correlation coefficient of MUIC and UIE was lower than that of creatinine-adjusted urine iodine (UICr) and UIE (0.575 vs 0.733), suggesting that after correcting for creatinine, UICr can reflect the iodine status better than MUIC. There was also significant difference among the median urine creatinine (MUC) in the trimesters.

### Serum concentration of thyroid function indices in different UIC groups

Table 6 depicts serum concentrations of FT3, FT4, TSH, SI, and Tg in different UIC groups. Compared with the 150–249 μg/L UIC group, Tg was significantly lower in the 250–499 μg/L UIC group. In contrast, serum SI was significantly higher in the 250–499 μg/L UIC group compared with the 150–249 μg/L UIC group. FT3, FT4 and TSH showed no significant differences between the UIC 150–249 μg/L group and the other UIC groups.

**Table 6:** Serum concentration of TSH, FT4, and Tg in different UIC groups

UIC group	N	FT3 (pmol/L)	FT4 (pmol/L)	TSH (mIU/L)	TG (μg/L)	SI (μg/L)	UIC(μg/L)
< 100	96(41.0%)	4.01 (2.88–5.03)	13.64 (10.05–18.53)	1.39 (0.06–3.29)	9.97 (8.85)	71.5 (45.25–111.88)	77.91 (32.02–98.97)
100–109	30(12.9%)	4.11 (3.34–7.02)	13.64 (10.18–24.98)	1.34 (0.01–2.63)	8.32 (5.64)	71.5 (49–118)	104.07 (100.68–110.34)
110–149	49(20.9%)	3.99 (3.21–5.07)	12.86 (9.35–16.67)	1.33 (0.14–3.59)	9.59 (8.80)	68 (44–101.75)	123 (111–149.24)
150–249	47(20.1%)	4.00 (3.21–5.25)	13.06 (9.08–17.60)	1.48 (0.12–2.93)	8.12 (9.91)	70 (39.8–105.8)	175.71 (150.05–237.39)
250–499	12(5.1%)	3.95 (3.29–9.87)	12.55 (11.04–22.14)	1.55 (0.01–2.88)	7.73 (3.85)^*^	78 (63–123)^*^	304.29 (259.00–471)

^*^P values represent the median level of this group compared with the 150–249 group

### Thyroid functions of newborn

All 79 pregnant women delivered their babies without complications, and boys accounted for 53.2% of the babies (Table 7). The average birth weight was 3310 g (3050–3600 g), and the average gestational age was 39 (38–40) weeks. Six of the newborns were preterm infants with gestational age less than 37 weeks. The average level of TSH from heel blood was 1.78 mIU/L (1.11–2.68 mIU/L). Five babies had TSH higher than 5 mIU/L, the highest being 7.3 mIU/L, which was still lower than the threshold of 10 mIU/L used as the screening cut-off value for clinical hypothyroidism.

**Table 7:** Basic information of the newborns

Indexes	N	Min	P25	P50	P75	Max
gestation	79	33	38	39	40	41
weight(g)	79	1510	3050	3310	3600	4370
TSH(mIU/L)	79	0.25	1.11	1.78	2.68	7.30

## Discussion

Our study results suggest that a region in which the USI target has been reached in the long term, the MUIC of pregnant women being slightly less than 150 μg/L is probably sufficient to maintain iodine related biological functions in both women and their newborns. A study by Andersson [13] also validated that where USI has been effective for at least 2 years (with salt adequately iodized and consumed by more than 90% [14] of the population), it can be expected that the iodine stored in the thyroid gland of pregnant women is sufficient to ensure adequate thyroid function.

Although MUIC is one of the common indices to assess iodine status of a population, the most important physiological function of iodine is to participate in the synthesis of thyroid hormones. So, the key point of this study was to evaluate whether an MUIC value below 150 μg/L will affect the thyroid function in pregnant women and their newborns and whether the current MUIC level (107.41 μg/L) can maintain normal thyroid function during pregnancy.

In this study, TgAb- and TPOAb-positive individuals were not enrolled in order to avoid the potential confounding factor of patients with thyroid autoimmune diseases. FT3, FT4, and TSH were used to evaluate the status of thyroid function throughout the pregnancy. In our results, the FT3 and FT4 initially declined and were then maintained. This could have been caused by the increasing basal metabolic rate and thyroid hormone consumption seen with the progression of a pregnancy. The trimester-specific changes we saw were in accordance with studies done by Soldin [15] and Kahric [16]. One woman in the study had an FT4 result slightly below the normal range and a TSH result a little over the normal range as defined in our study. But when compared to the reference range used by Donnovan et al. [17], lab values were all in the normal range. In this subject, the indicators returned to reference range by the 32nd week without any treatment. Furthermore, the Tg and SI were within the normal range during the whole pregnancy, and her newborn’s TSH was less than 5 mIU/L, which could indicate the nutritional status of the mother. After consulting the endocrinologist who mainly diagnosed on the basis of experience and the blood tests, we determined that this subject did not need immediate medical intervention and could still be included in our analysis.

Tg was also detected across all 3 trimesters. The values of Tg and TSH both had a “U” shape tendency during the whole pregnancy, which could be affected by human chorionic gonadotropin (HCG). HCG can have a thyroid stimulating effect [18]. It can competitively inhibit the excretion of TSH [19] and cause the increase of Tg [20]. Some studies [21, 22] indicate that if iodine deficiency exists in the body, the concentration of Tg will increase correspondingly, and the change of Tg concentration is more sensitive than that of goiter. Although there is no consensus for the reference intervals of Tg during pregnancy, ≥ 13 μg/L is commonly used when assessing iodine deficiency in pregnant women [23]. In our study, serum Tg levels were negatively correlated with urinary iodine level (r = − 0.14). Mild to moderate iodine deficiency may be associated with elevated serum Tg. Tg levels of our participants in all 3 trimesters were less than 13 μg/L, and the median Tg was 11.8 μg/L. According to Moleti et al. [24], the median Tg level was 10.2 μg/L which is similar to the finding in our study.

Serum iodine was another biomarker used to monitor the iodine status of our volunteers. This reflects the level of bioactive iodine ions that can be utilized by thyroid tissue in vivo [25]. It was reported as a relatively stable index and not able to be readily changed with diet [25]. The correlation analysis also shows a strong positive relationship between SI and FT4, especially at 32 weeks’ gestation (r = 0.734, *P* < 0.05). Similar to Tg, there is also no unified threshold for SI. The recommended reference range from the WHO, the Mayo Clinic, and other studies is 45–90 μg/L, 52–109 μg/L, and 36–97 μg/L, respectively. The distribution of SI in our study was 44–109 μg/L.

The serum concentrations of thyroid function indexes were compared in different UIC groups. The 150–249 μg/L UIC group, the range recommended by the WHO, was used as the reference group. Except for Tg and SI in the 250–499 μg/L UIC group, there was no significant difference by UIC level. Thus, our hypothesis suggests that iodine intake in early pregnancy could be UIC less than 150 μg/L. These findings were consistent with the results in the lower iodine intake groups of Xiaoguang Shi [26].

Dietary iodine intake is an important index for evaluating iodine nutritional status. Our results showed that the average total dietary iodine intake in this study was 231.17 μg/d. This value reached the recommended nutrient intake (RNI) of iodine for Chinese pregnant women (230 μg/d) [27].

Dietary iodine intake fluctuates greatly according to what the individual ate before antenatal care, so after each visit and collection of the dietary intake log, we provided feedback about the results and gave professional advice about her dietary intake. Our study subjects knew more about the important role and the additional requirement of iodine in pregnancy after receiving our training during the study. The majority reported that they returned to buying iodized salt for their family again.

MUIC has been widely used to assess the iodine status of a population [28]. According to urinary iodine excretion accounted for 90% of total iodine excretion [29, 30], 200.11 μg/d UIE means the 222.34 μg/d iodine loses per day. Compared with the total dietary iodine intake was 231.17 μg/d (Table 4), the urinary losses suggest pregnant women in our study were in a positive balance of iodine metabolism during the whole pregnancy. These two methods for evaluating the iodine intake status both indicated that subjects in our study had good dietary iodine intake.

All the data suggest that the pregnant women in our study have the appropriate iodine nutritional status. Their median UIC during the whole gestation period was about 110 μg/L, with no significant differences among trimesters.

Neonatal heel blood TSH was another recommended indicator for iodine status by WHO/UNICEF/ICCIDD [4]. A TSH value of 10 mIU/L is widely used as the screening cut-off for clinical hypothyroidism. The WHO also points out that less than 3% of neonatal heel blood TSH > 5 mIU/L could indicate overall iodine sufficiency in a region [5, 31]. Our study had 5 babies (6.3%) who had a heel blood TSH higher than 5 mIU/L. Although the percentage is higher than 3%, all the detection values were lower than the 10 mIU/L cut-off. Some studies [32, 33] found that when screening neonatal TSH areas with sufficient iodine, many regions had a frequency of TSH > 5 mIU/L higher than 3%. Although several studies have attempted to find the influence of iodine deficiency on child neurobehavioral development, there are no data available from any randomized controlled clinical trials linking prenatal iodine supplementation and child neurobehavioral development in regions of mild to moderate iodine deficiency [34].

Taking all this information together, we conclude that all the pregnant women in our cohort study had good thyroid function across trimesters.Our study suggests that in China, high and long-term coverage with adequately iodized salt might justify a different cut-off for MUIC, especially for pregnant women.

A major strength of this study is that we collected 24-h urine samples during all trimesters and analyzed it for iodine and creatinine at the same time to correct the effects of incomplete sample collection. The sodium content of the 24-h urine collection was also used to get more accurate salt intake information in this study. Although a morning spot urine was used for screening at 6 weeks, the WHO/UNICEF/ICCIDD joint task force indicates that iodine concentrations measured in urine samples collected in the morning, or from other spot urine collections, have been shown to adequately assess a population’s iodine status [4, 35]. Therefore, the collected urinary samples are representative during the whole gestation. Second, thyroid function during pregnancy was monitored throughout gestation, even extending to the newborn’s heel blood TSH. Besides the general indictors, Tg and SI were also used to assess thyroid function. We excluded individuals with positive TPOAb and TgAb. Third, detailed dietary iodine intake from foods, drinking water, soup, and salt were analyzed in our study. Positive iodine metabolism balance gives us more confidence about our results.

However, this study does have some limitations. For the limited time and budget, our sample size of 79 is relatively small. Further multi-centered clinical research is needed to verify our results. Another limitation is lack of clarity about the iodine in salt used to make processed foods. Relevant research of iodine concentration in processed food is underway.

## Conclusions

In this study, with over 20 years implementing of universal salte iodization in China, the expectant mothers with the MUIC 107.4 μg/L, which was less than 150 μg/L recommended by WHO, can also maintain the normal iodine nutritional status for both of the pregnant women and their newborn babies.

## Data Availability

Please contact author (Lichen Yang) for data or material requests.

## Abbreviations

FT3: Free Triiodothyronine
FT4: Free Thyroxine
MUC: median urine creatinine
MUIC: median urinary iodine concentration
SI: serum iodine
TG: thyroid globulin
TGAb: thyroglobulin antibody
TPOAb: thyroid peroxidase antibody
TSH: Thyroid Stimulating Hormone
UCE: median urine creatinine excretion
UICr: creatinine-adjusted urine iodine
UIE: urinary iodine excretion
USI: universal salt iodization
V24 h: 24-h urine volume
